# Clinical recognition, management, and research progress of infantile perianal protrusion: a narrative review and literature-based case aggregation

**DOI:** 10.3389/fped.2026.1958437

**Published:** 2026-10-09

**Authors:** Lintao Liu, Liyun Xia, Shuangai Liu, Weili Xu

**Affiliations:** Department of Pediatric Surgery, The Second Hospital of Hebei Medical University, Shijiazhuang, China

**Keywords:** constipation, dermoscopy, infantile perianal pyramidal protrusion, perianal (perineal) protrusion, perianal lesions

## Abstract

**Background:**

Infantile perianal protrusion (IPP) is a benign clinical lesion or phenotype, usually arising from or adjoining the anal verge and occurring predominantly in girls. Although encountered in pediatric practice, it remains underrecognized and may be mistaken for other anal or perianal abnormalities, resulting in unnecessary investigations or treatment. The evidence consists mainly of case reports and small case series.

**Methods:**

We conducted a narrative review with literature-based case aggregation. A systematic search of PubMed/MEDLINE, Embase, Web of Science, the Cochrane Library, ProQuest, and Scopus was performed from database inception to 30 October 2025, using the principal and historical terms for this lesion, including *infantile perianal protrusion*, *infantile perianal pyramidal protrusion*, *infantile perineal protrusion*, *perineal protrusion*, *perineal nodule*, *perianal skin tag*, *anal skin tag*, and *anal fold*, along with their spelling and plural variants. Reference lists of eligible articles were also screened. For included reports, we extracted and analysed data on nomenclature, demographic characteristics, lesion morphology and location, associated conditions, proposed pathogenesis, histopathological and dermoscopic features, management, and outcomes. Potentially overlapping cases were identified on the basis of authorship, institution, recruitment period, and case characteristics, and were counted only once when duplication was likely.

**Results:**

The literature search identified 169 published patients, comprising 163 children and 6 adult women, with a marked female predominance. Lesions were usually solitary and most commonly located at the anterior anal midline, though posterior, lateral, multiple, and circumferential presentations were also reported. Three subtypes were recognized: constitutional, acquired functional, and lichen sclerosus-associated. Constitutional lesions were typically asymptomatic and often self-resolving. Acquired functional lesions were frequently associated with constipation or local irritation and improved after management of contributing factors. Lichen sclerosus-associated lesions showed characteristic histopathological changes. In non-lichen sclerosus cases, histopathology was generally nonspecific, whereas dermoscopy revealed a recurrent vascular pattern in reported cases.

**Conclusions:**

IPP is usually a benign anal-verge/perianal clinical lesion with a favorable course. Typical lesions can generally be recognized clinically and managed with reassurance and observation, without routine biopsy, imaging, or excision. Associated conditions—particularly constipation, local irritation, fissuring, dermatitis, fistula, or lichen sclerosus—should be assessed and treated when suggested by the history or examination rather than investigated routinely in every patient. Greater awareness may reduce misdiagnosis and unnecessary intervention.

## 1 Introduction

Infantile perianal protrusion (IPP) is a benign clinical lesion or phenotype observed predominantly in infants and young children, especially girls. In its typical form, it arises from, or is continuous with, the anal verge at the anterior midline and projects toward the perineum. Less commonly, similar protrusions have been described at the posterior or lateral anal verge, at multiple sites, or with extension into the adjacent perianal or vulvar skin. Accordingly, “perianal” is used in this review as a pragmatic umbrella term derived from the existing literature, although “anal-verge protrusion” may be anatomically more precise for many typical lesions. The condition was first recognized in 1989, when McCann et al., in a study of normative anogenital anatomy in prepubertal children without a history of sexual abuse, described characteristic acrochordon-like protrusions along the anal midline in 18 girls, most of which were located anterior to the anus (1). These lesions were referred to as “anal skin tags/folds”, and a congenital origin was suggested. In 1996, Kayashima et al. (2) reported 15 similar cases and introduced the term infantile perianal pyramidal protrusion (IPPP) on the basis of the lesion's typical pyramidal appearance. Since then, additional cases have been reported worldwide, and a variety of terms—including infantile perineal protrusion, perineal nodule, and infantile perianal protrusion—have been used in the literature, reflecting the gradual evolution of knowledge regarding this entity. Because the lesion is not invariably pyramidal and is not always confined to the perineal raphe, the term “infantile perianal protrusion” may better encompass its clinical spectrum.

Although IPP is benign and likely not rare in pediatric practice, it remains insufficiently recognized by clinicians. Most patients present with a small, smooth, soft-tissue protrusion in the anterior perianal region, but the lesion may also appear posteriorly, laterally, or, in rare cases, at multiple sites. Because of its variable morphology and the limited awareness of this condition, IPP is frequently misdiagnosed or overlooked. It may be confused with hemorrhoids, condyloma acuminatum, rectal prolapse, sentinel tags associated with fissure, Crohn's disease, traumatic lesions, or even findings suspicious for sexual abuse. Such diagnostic uncertainty may result in unnecessary investigations, inappropriate treatment, avoidable specialist referral, and considerable anxiety for caregivers. Improving familiarity with IPP is therefore clinically important, particularly for pediatricians, pediatric surgeons, dermatologists, and other clinicians involved in the evaluation of perianal lesions in children.

To date, the evidence base for IPP remains limited, consisting almost exclusively of case reports and small case series, with only slightly more than one hundred patients described in the literature. Consequently, many important issues remain incompletely understood, including the true incidence of the condition, its pathogenesis, the significance of its female predominance, the relationship between constitutional and acquired forms, the role of constipation and local inflammation, and the association between IPP and lichen sclerosus or congenital anomalies. Histopathologic and dermoscopic descriptions are also sparse, and management strategies have largely been guided by clinical experience rather than robust evidence. In this review, we summarize and analyze the literature on IPP from the past three decades, with a focus on its nomenclature, epidemiology, clinical features, pathogenesis, histopathology, dermoscopy, differential diagnosis, management, and prognosis, in order to improve recognition of this underreported condition and support clinical decision-making.

## 2 Nomenclature and anatomical definition

The terminology applied to this lesion has evolved alongside changing interpretations of its morphology and anatomical origin. In 1996, based on the characteristic clinical features of 15 cases, Kayashima et al. (2) introduced the term *infantile perianal pyramidal protrusion* (IPPP). The lesions were typically pyramidal, smooth surfaced, red to rose-pink, and located at the anterior anal midline. In 1998, Cruces et al. (3) reported four anterior midline protrusions associated with lichen sclerosus et atrophicus, now generally termed lichen sclerosus. They noted that the lesions were not invariably pyramidal and argued that “perineal” more accurately reflected their location along the perineal raphe. They therefore proposed the simpler term *infantile perineal protrusion*. This view was supported by Konta et al. (4), who reported 36 perineal nodules in 2000, all located along the anterior perineal midline and none of which were pyramidal.

Subsequent reports, however, demonstrated greater anatomical variation. In 2002, Patrizi et al. (5) described 13 pediatric cases, including three patients with lesions posterior to the anus and two with both anterior and posterior lesions. Because the lesions were not invariably confined to the anterior perineal raphe, the authors considered “perianal” a broader descriptor and proposed the term *infantile perianal protrusion* (IPP). Later descriptions of lateral, multiple, and circumferential lesions further supported the use of a less morphologically and anatomically restrictive umbrella term. Moreover, similar lesions are not confined to childhood. In 2015, Gorcey et al. (6) reported six adult women with perianal protrusions and suggested that some lesions may have been present since childhood but failed to regress. Similar protrusions had been described even earlier: in 1978, Wienert et al. (7) reported lesions in both infants and older adults under the designation “perianal skin tags.”

The different terms used in the literature partly reflect distinct anatomical concepts. The anal verge is the junctional margin surrounding the external opening of the anal canal; the perianal skin refers to the surrounding keratinized skin; and the perineal midline or raphe extends anteriorly from the anus toward the external genitalia. Review of the available clinical descriptions and images suggests that many typical lesions arise directly from, or are continuous with, the anal verge, particularly at the anterior midline, and may extend toward the perineal raphe. They should therefore not necessarily be regarded as primary lesions of the surrounding perianal skin. Posterior and lateral lesions also commonly appear to be anatomically related to the anal verge, although the precise site of origin cannot be determined from every published report.

The adjective “pyramidal” is clearly too restrictive because the reported lesions may be leaf-like, tongue-like, crest-like, elongated, polypoid, or otherwise non-pyramidal. Nevertheless, neither “perineal” nor “perianal” precisely describes every reported anatomical configuration. Although anal-verge protrusion may be anatomically more accurate for the typical lesion, we use *infantile perianal protrusion* as a pragmatic umbrella term in this review because it is broadly established in the literature and encompasses anterior, posterior, lateral, and multiple presentations. The term “infantile” is also retained for historical consistency, while recognizing that persistent or analogous lesions have occasionally been reported in adults. Future reports should describe explicitly whether a lesion arises from the anal verge, adjacent perianal skin, or perineal raphe rather than relying solely on a historical diagnostic label.

We regard IPP as a descriptive clinical lesion or phenotype rather than a single disease entity. It may occur as a primary congenital or idiopathic finding or as a secondary change associated with local mechanical, anorectal, inflammatory, or dermatologic conditions, including constipation, straining, fissuring, irritation, dermatitis, and lichen sclerosus. In the published literature included in our case aggregation, 169 patients have been described, including 163 children and six adult women, under labels such as IPPP, infantile perineal protrusion, perineal nodule, and anal or perianal skin tag/fold (Table 1). This total represents an aggregation of published cases and should not be interpreted as an estimate of population frequency or geographic distribution.

**Table 1:** Summary of published articles on infantile perianal protrusion to date.

No.	Author	Year of publication	Country	Male/Female	Age at diagnosis
1	McCann et al. (1)	1989	USA	–/18	2 months–11 years
2	Kayashima et al. (2)	1996	Japan	1/14	1–30 months
3	Merigou et al. (8)	1998	France	–/4	6–20 months
4	Cruces et al. (3)	1998	Spain	–/4	2–7 years
5	Konta et al. (4)	2000	Japan	–/36	0–11 years
6	Patrizi et al. (5)	2002	Italy	–/13	7 months–7 years
7	Miyamoto et al. (9)	2004	Japan	–/1	11 months
8	Fleet et al. (10)	2005	USA	–/1	12 months
9	Khachemoune et al. (11)	2006	Caucasian^a^	–/2	4–9 months
10	Hernandez et al. (12)	2007	Spain	–/1	11 years
11	Kim et al. (13)	2007	Korea	–/1	19 months
12	Chute et al. (14)	2009	USA	–/1	3 months
13	Leung et al. (15)	2010	Canada	–/2	9–11 months
14	Haastrup et al. (16)	2011	Denmark	–/1	13 years
15	Zavras et al. (17)	2012	Greece	2/6	6 months–13 years
16	Cavicchioli et al. (18)	2014	Italy	–/2 (twins)	3 h
17	Verma et al. (19)	2014	India	–/2	2–3 months
18	Gungor et al. (20)	2014	Turkey	–/1	9 months
19	Song et al. (21)	2014	Korea	–/1	2 years
20	Ferrari et al. (22)	2015	Argentina	2/9	0–7 years
21	Lamberti et al. (23)	2015	Italy	–/1	9 months
22	Scaparotti et al. (24)	2017	USA	–/1	10 months
23	Sharma et al. (25)	2018	India	–/1	4 months
24	Adnan et al. (26)	2021	USA	–/1	0
25	Zhang et al. (27)	2023	China	–/1	2 years
26	Dhami et al. (28)	2023	USA	3/24	0–4 years
27	Bartolomeo et al. (29)	2023	Italy	–/6	1 months–8 years
28	Gorcey et al. (6)	2015	USA	– 6 (adult)	20–68 years

a “Caucasian” as reported in the original paper.

## 3 Demographic characteristics and frequency in published reports

Excluding the six adult women, the published literature identified 163 pediatric patients aged from birth to 13 years, including 155 girls and 8 boys. Thus, girls accounted for 95.1% of the aggregated published pediatric cases. This marked female predominance has been consistently described; however, because the evidence is derived mainly from case reports, small case series, and selected clinical cohorts, this proportion should not be interpreted as a population-based sex ratio or comparative risk estimate.

Two observational studies provide limited information on the frequency with which these lesions were identified in specifically examined pediatric cohorts. McCann et al. (1) observed anal skin tags/folds in 18 of 164 prepubertal girls examined for normative anogenital anatomy (11.0%), whereas Konta et al. (4) identified perineal nodules in 36 of 500 children (7.2%), all of whom were girls. These figures represent the proportions detected within the respective study populations and examination settings. They do not establish the incidence or population prevalence of the lesion because the cohorts were selected, the case definitions and ascertainment methods differed, and the findings have not been confirmed in representative population-based studies.

Published pediatric cases originated from several countries, with the largest numbers reported from Japan, the United States, and Italy. These publication counts should not be interpreted as evidence of true geographic differences in disease frequency. They are likely to reflect variation in clinical awareness, terminology, examination practices, referral patterns, research interest, and publication or reporting bias. The true population frequency and geographic distribution therefore remain unknown.

Overall, the available literature suggests that this lesion may be encountered more often in pediatric practice than the limited number of publications implies, but this impression remains unquantified. Population-based studies using a standardized anatomical definition and consistent case-ascertainment methods are needed to determine its prevalence, sex distribution, and natural history.

## 4 Clinical features

IPP typically manifests as a soft-tissue protrusion around the anus. Details are as follows:

### 4.1 Location and anatomical origin

Most published lesions were solitary and involved the anterior midline. Where clinical photographs or sufficiently detailed descriptions were available, the typical lesion appeared to arise from, or remain continuous with, the anterior anal verge and to project toward the perineal midline. Less commonly, lesions involved the posterior or lateral anal verge, occurred at more than one site, extended into adjacent perianal or vulvar skin, or encircled part or all of the anal opening. Because many reports used “perianal,” “perineal,” and “anal” interchangeably, the exact tissue of origin could not always be determined retrospectively. Future reports should distinguish lesions arising from the anal verge from those arising independently in the surrounding perianal skin.

### 4.2 Shape

Morphology varies. The classic form is pyramidal; other descriptors include leaf-like, hen's-crest-like, tongue-tip-like, peanut-like, cigar-like, and stalked. Patrizi et al. (5) reported 13 new IPP cases and observed that acquired lesions were pyramidal whereas congenital lesions were leaf-like. Our findings do not support a necessary correlation between subtype and external configuration. Subtyping criteria were as follows:

1. Acquired (functional) type: secondary to atopic dermatitis, constipation, diarrhea, anal fissure, perianal fistula, irritant diaper dermatitis, congenital rectovaginal fistula, rectovestibular fistula, etc.
2. LS-associated IPP: suspected or confirmed clinical features of lichen sclerosus (LS) with histopathologic confirmation when available.
3. Constitutional type: including congenital cases (present at birth) and other cases without secondary factors.

### 4.3 Symptoms and proposed practical classification

The clinical presentation of IPP varies according to whether the lesion occurs in isolation or in association with a local mechanical, inflammatory, anorectal, or dermatologic condition. For practical clinical purposes, we propose a simplified descriptive classification into primary and secondary IPP. This framework replaces the previous categories of constitutional, acquired functional, and lichen sclerosus–associated IPP.

1. Primary IPP: Primary IPP includes congenital lesions present at birth and idiopathic lesions for which no contributing local mechanical, inflammatory, anorectal, or dermatologic condition can be identified. These lesions are usually asymptomatic and may remain stable, regress spontaneously, or occasionally persist into later life.

The subsequent development of constipation, anal fissure, or local irritation does not necessarily establish a secondary origin, particularly when the protrusion was clearly present before the associated condition. Nevertheless, some apparently primary lesions have enlarged following diarrhea or the passage of hard stools (25, 28). In one reported patient, a protrusion appeared suddenly within 1 h after the passage of a hard stool (23). These observations suggest that mechanical factors may precipitate or accentuate the lesion in some patients, although they do not establish a uniform mechanism. The distinction between primary and secondary IPP may therefore be uncertain when the temporal sequence is incompletely documented.

2. Secondary IPP: Secondary IPP refers to lesions arising in temporal or clinical association with a potentially contributory local condition. Reported associations include constipation, repetitive straining, painful defecation, passage of hard stools, diarrhea, anal fissure, irritant diaper dermatitis, atopic dermatitis, perianal fistula, congenital rectovaginal fistula, rectovestibular fistula, and lichen sclerosus (3, 28). Symptoms in these patients generally reflect the associated condition rather than the protrusion itself and may include constipation, pain or straining during defecation, bleeding from the lesion or blood in the stool, local irritation, and weeping.

In a retrospective series of 27 patients with IPP, Dhami et al. (28) reported constipation in 17 patients, straining or pain during defecation in three, bleeding from the lesion or blood in the stool in three, and weeping from the lesion in one; seven patients had more than one symptom. Constipation was therefore the most frequently reported association in that series. However, because the study was retrospective and lacked an unaffected comparison group, these findings do not demonstrate that constipation caused the lesions.

#### 4.3.1 Lichen sclerosus as an important secondary association

Lichen sclerosus should be considered when an anal-verge protrusion is accompanied by porcelain-white or hypopigmented plaques, atrophy, sclerosis, ecchymosis, fissuring, erosions, pruritus, pain, dysuria, or persistent constipation. In this setting, the protrusion may represent a secondary morphological change within the spectrum of anogenital lichen sclerosus rather than a distinct IPP subtype. Histopathologic confirmation may be considered when the clinical diagnosis is uncertain, although biopsy is not routinely required for a typical uncomplicated primary lesion.

The previously used term *lichen sclerosus et atrophicus* has largely been replaced by *lichen sclerosus*, because epithelial atrophy is not present in every case and some lesions exhibit epithelial hypertrophy (30). Lichen sclerosus is a chronic inflammatory and potentially scarring dermatosis with a predilection for anogenital skin. Typical clinical findings include porcelain-white or hypopigmented plaques, skin atrophy or sclerosis, ecchymoses, fissuring, excoriations, erosions, and, less commonly, bullae (31). Common symptoms include anogenital pruritus, soreness, pain, dysuria, and constipation, particularly in children (32).

In a review by Balakirski et al. (33) of 4,516 pediatric cases of lichen sclerosus, detailed clinical information was available for 1,046 girls and 2,743 boys. Hypopigmentation and skin atrophy were frequent findings in girls, whereas boys more commonly exhibited scarring or alterations in genital anatomy. Symptoms such as pruritus, pain or burning, dysuria, and constipation were reported more frequently in girls. These findings describe pediatric lichen sclerosus generally and should not be interpreted as estimates of the frequency of IPP among children with lichen sclerosus.

During our review, protrusion-like findings at the anterior anal midline were visible in figures from some publications on lichen sclerosus but were not discussed in the accompanying text (34, 35). Because a diagnosis cannot be established reliably from published images alone, these observations were not included as additional IPP cases in the case aggregation. They nevertheless suggest that future studies of pediatric anogenital lichen sclerosus should document anal-verge morphology systematically.

When IPP is accompanied by hypopigmentation, porcelain-white plaques, atrophy, sclerosis, ecchymosis, fissuring, erosions, persistent pruritus, pain, or dysuria, careful evaluation for lichen sclerosus is warranted. Management should primarily address the associated dermatosis, and appropriate clinical follow-up may be required. By contrast, routine investigation for lichen sclerosus is not indicated in every patient with an otherwise typical, asymptomatic protrusion and no supportive clinical findings.

#### 4.3.2 Classification limitations

This primary–secondary classification is intended as a pragmatic clinical framework rather than a validated etiologic system. In many published cases, the temporal relationship between IPP and associated factors was not adequately documented. Mechanical, inflammatory, and dermatologic conditions may cause a lesion, promote enlargement of a pre-existing lesion, or simply coexist with it. Accordingly, classification should be based on the chronology and overall clinical context, and causality should not be inferred solely from the presence of an associated condition. The definitions, clinical features, associated conditions, and general management of these two categories are summarized in Table 2.

**Table 2:** Classification, clinical features, and general management of IPP.

Classification	Definition	Clinical features and associations	General management
Primary IPP	Congenital or idiopathic lesion without an identifiable contributing local condition	Usually asymptomatic; may remain stable or regress; may occasionally enlarge after local mechanical irritation	Reassurance and observation
Secondary IPP	Lesion temporally or clinically associated with a potentially contributory condition	Constipation, straining, hard stools, diarrhea, fissure, dermatitis, irritation, fistula, or lichen sclerosus	Treat the associated condition; lesion-specific intervention is rarely required

#### 4.3.3 Reported associations with congenital anomalies

A small number of reports have described IPP-like lesions in patients with congenital anomalies. In 2006, Girard et al. (36) introduced the acronym PELVIS syndrome to describe the association of perineal hemangioma, external genital malformations, lipomyelomeningocele, vesicorenal abnormalities, imperforate anus, and skin tag. In 2014, Verma et al. (19) described two patients with IPP-like protrusions and perineal or perianal hemangiomas and interpreted these presentations as incomplete PELVIS syndrome, with the “skin tag” component manifesting as IPP.

In the retrospective series of 27 patients reported by Dhami et al. (28), one child had a congenital rectovaginal fistula, one had a sacral cleft with occult spina bifida, and one had growth retardation. The authors regarded these findings as coincidental rather than directly related to IPP. Given the small number of cases, heterogeneous abnormalities, and absence of comparative data, current evidence does not support considering IPP a marker of congenital malformation or routinely screening children with a typical isolated lesion for underlying anomalies. Additional assessment should instead be guided by the presence of hemangiomas, abnormal sacral or neurologic findings, atypical anorectal anatomy, urinary abnormalities, or other relevant clinical features.

#### 4.3.4 Reported associations with congenital anomalies

A 9-year-old girl presented with a rectovestibular fistula that had been recognized for more than 3 years. Her medical history was otherwise unremarkable, with no relevant family history. Physical examination revealed an approximately 2.0-mm fistulous opening in the vaginal vestibule, with intermittent passage of loose stool through the opening. Digital rectal examination revealed no additional abnormalities. A pink, soft, pyramidal-shaped protrusion was also observed on the right side of the anus (Figure 1). According to the family, the perianal protrusion had been noticed at approximately the same time as the rectovestibular fistula. The patient was clinically diagnosed with a rectovestibular fistula accompanied by an infantile perianal protrusion. Transanal repair of the rectovestibular fistula was performed without complications, and the patient was discharged 2 weeks after surgery. At the 6-month follow-up, the perianal protrusion remained present and showed no significant change from its preoperative appearance.

**Figure 1:**
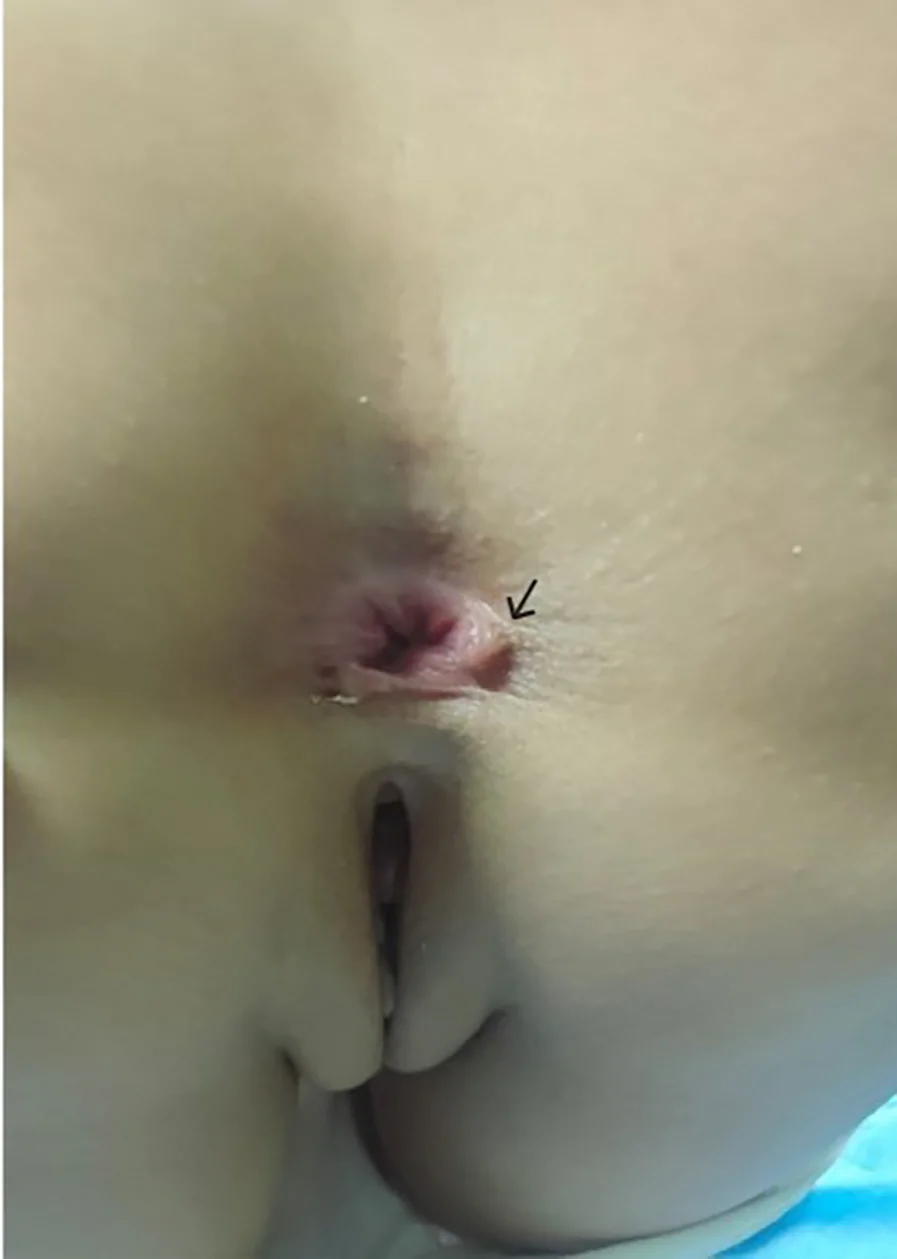
A lateral perianal protrusion in a child with a rectovestibular fistula. A pink, soft, pyramidal-shaped protrusion is visible on the right side of the anus. The lesion was clinically considered an infantile perianal protrusion associated with a rectovestibular fistula.

## 5 Pathogenesis

The pathogenesis remains unclear, with multiple hypotheses:

### 5.1 Mechanical stimulation related to wiping/defecation

Kayashima et al. (2), who first defined the disease, postulated that mechanical irritation from wiping after defecation leads to lesion enlargement—a view widely supported. Khachemoune (11) similarly hypothesized that IPP represents outward protrusion of near-normal redundant skin secondary to physical irritation. Objections include: (1) Merigou (8) did not find increased irritation from post-defecation wiping; (2) This hypothesis does not account for occurrence in neonates/infants whose stools are softer than in older children, although diarrhea, fistulae, and fissures indicate genuine perineal mechanical stresses (17); (3) In Konta's (4) examination of 500 children, 36 girls had such nodules. Among 276 girls analyzed by age, neonates had the highest prevalence (15.8%, 6/38). Since older children are more prone to hard stools, a wiping-induced mechanism would predict higher prevalence in older groups, which was not observed. Nevertheless, biopsy in four constitutional cases suggested IPP may be near-normal redundant skin protruding outward: three (two present at birth; one detected at 6 months) showed essentially normal epidermis/dermis, while one case (present at birth, enlarged after passing a hard stool) exhibited acute inflammation.

### 5.2 Constitutional weakness of the female perineum

The perineum lies within the osteoligamentous framework of the inferior pelvic outlet, with rhomboid contour bounded anteriorly by the inferior pubic symphysis, laterally by the ischial tuberosities, and posteriorly by the coccyx. A transverse line between the anterior ischial tuberosities divides it into two triangles: a smaller anterior urogenital region and a larger posterior anal region. While the anal region is similar between sexes, the urogenital region differs substantially. The perineum is a musculofascial structure closing the pelvic floor inferiorly, with weak points centrally and laterally manifested as inter-muscular gaps (23, 37). Anatomically, constitutional IPP may arise from these weak points in the pelvic floor/perineum, explaining the female predominance. Clinical observations support this view: sudden IPP appearance 1 h after passing a hard stool (9); congenital cases enlarging after hard stool (4); and similar abnormalities in elderly women (8). Moreover, intermittent constipation observed in acquired IPP cases also supports this hypothesis (8, 10, 11, 15, 17, 21, 23, 24, 27).

### 5.3 Embryologic remnant hypothesis

The perineum forms during fetal development by elongation of the urorectal septum. As IPP often occurs along the perineal midline, it may represent a residual prominence of the apex of the urorectal septum (4, 11). Of 163 pediatric cases, 33 (20.2%, 33/163) were present at birth. This hypothesis accounts for congenital IPP and suggests a constitutional trait.

### 5.4 Visibility in females

One early notion was that IPP is simply more visible in females, hence seemingly more common (2). This is untenable. Although early underrecognition—especially in boys—could have led to missed diagnoses, more recent reports (2014–present) include only 5 boys among 55 children, despite increased awareness and more careful perineal examination in boys. We therefore consider the female predominance to reflect true disease biology.

### 5.5 LS-related fibrotic remodeling

LS-associated IPP may result from fibrous tissue reorganization due to inflammation (5). Two comments: ① Besides LS, other acquired conditions—atopic dermatitis, diarrhea, anal fissure, fistula, irritant diaper dermatitis, congenital rectovaginal or rectovestibular fistula—also induce perianal inflammation; could the same mechanism underlie acquired IPP? ② Given that constipation occurs in 14.9% of girls with LS (33), might IPP, in some cases, be secondary to constipation rather than directly caused by LS?

### 5.6 Genetic predisposition

Ten patients (6.1%, 10/163) had a clearly documented family history, likely an underestimate given incomplete reporting. Of these, six (60%, 6/10) were present at birth (5, 17, 18)—including a pair of monoamniotic monochorionic twins—suggesting heritability. Two were LS-associated and were sisters (3); LS itself has genetic underpinnings, with one large study reporting a 12% family history rate (38), implying that heritability in LS-associated IPP may reflect LS susceptibility with IPP secondary to LS. One case was acquired (constipation-related) and the family history may be incidental (22). The final case had anal fissure and anogenital erythema and could be acquired or LS-associated; no biopsy was performed, so subtype remained uncertain (5).

## 6 Histology and dermoscopy features

### 6.1 Histology

Due to parental refusal of biopsy and limited clinician awareness, only a small subset (15/163, 9.2%) underwent histopathology. Seven exhibited classic features diagnostic of LS (3, 5, 12, 13). Definitive LS diagnosis relies on skin biopsy from the whitest, most indurated area; histopathology is characteristic across ages: typically epidermal atrophy, basal cell vacuolar change, a homogenized pale zone in the upper dermis, and variable lichenoid mononuclear infiltrate in the mid-dermis (39). We also note that in two biopsied cases described by Kayashima et al., the histology and images strongly supported LS, in line with Cruces' findings (3).

In four constitutional IPP cases, Konta et al. (4) reported essentially normal epidermis and dermis, with focal acanthosis, thickening and/or elongation of rete ridges, capillary dilation or congestion, and dilated lymphatics; when lesions had recently enlarged, sparse neutrophils and eosinophils were present in the superficial dermis. Song et al. (21) reported an acquired, constipation-related IPP with epidermal acanthosis and a fibrovacular core with lymphocytic infiltration. Overall, these findings suggest that—except when LS is present—constitutional and constipation-related acquired IPP show non-specific or near-normal histology, resembling a “skin tag.”

### 6.2 Dermoscopy

Dermoscopy is a simple, noninvasive method that reveals superficial morphologic features not visible to the naked eye. Lamberti (23) and Bartolomeo (29) performed dermoscopy in 1 acquired case and 6 IPP cases (1 congenital, 1 acquired, 1 LS-associated, and 3 unspecified), respectively. They consistently observed a recurrent vascular pattern over the lesion: multiple rows of globular, dotted, or linear vessels aligned along the main axis of the protrusion, corresponding to variably dilated capillaries. The three IPP subtypes shared this dermoscopic vascular signature, which may be a characteristic attribute of IPP and deepens our understanding of the condition. Because dermoscopic findings have been described in only a small number of cases, they should be regarded as supportive rather than diagnostic.

## 7 Management and prognosis

For constitutional IPP, including congenital and familial cases, conservative observation is sufficient. Resolution has been documented as early as 3 days (26), within 6 weeks (18), or within 6 months (20). In four constitutional cases followed by Konta (4) for 3, 2.5, 2, and 6 years after initial evaluation, all lesions decreased over time; one congenital lesion resolved completely after 3 years. However, some congenital IPP persisted up to 11 years without resolution (5). Prognosis in constitutional IPP is clearly individualized, and observation is appropriate. Notably, when a congenital IPP completely covers the anus, it can be misdiagnosed as imperforate anus (26). There is also a report of childhood capillary hemangioma presenting as Infantile Perianal Protrusion (40). Careful physical examination is essential for differentiation.

For acquired IPP, treat the underlying condition. With constipation management, IPP may decrease or resolve (8, 10, 11, 15, 17, 23, 24, 27), although persistence also occurs (5, 22, 29). Regardless of outcome, treatment of the precipitating condition is beneficial.

For LS-associated IPP, when LS is suspected, actively seek supporting evidence and perform biopsy if necessary, because vulvar lichen sclerosus (VLS) requires accurate diagnosis and proactive treatment. Without adequate therapy, VLS can cause severe scarring, atrophy and deformation of the vulva, and narrowing of the introitus, impairing quality of life. Moreover, it is a risk factor for vulvar squamous cell carcinoma, with a lifetime risk of 2%–6% in untreated or undertreated disease (39). Early diagnosis and proper treatment of LS are crucial (35).

## 8 Conclusions

Infantile perianal protrusion is best regarded as a descriptive clinical lesion or phenotype, typically arising from or adjoining the anal verge, rather than as a single disease entity. Most typical lesions are benign, asymptomatic, and self-limited or stable. Recognition of the characteristic morphology and anatomical relationship to the anus is usually sufficient for diagnosis, and routine biopsy, imaging, excision, or extensive laboratory investigation is not warranted.

Clinical assessment should remain directed by the individual presentation. Constipation, straining, fissure, dermatitis, local irritation, fistula, and signs of lichen sclerosus should be sought and managed when indicated by the history or examination. Atypical, ulcerated, bleeding, rapidly changing, persistent, or diagnostically uncertain lesions may require specialist evaluation or targeted investigation.

A practical distinction between primary congenital/idiopathic lesions and secondary lesions associated with local mechanical, inflammatory, anorectal, or dermatologic factors may facilitate management, although this framework requires further validation. Future studies should use standardized descriptions of the lesion's origin at the anal verge, perianal skin, or perineal raphe and should investigate population frequency, natural history, and associated conditions prospectively.
